# Treatment with ropeginterferon alfa-2b in patients with hydroxyurea resistant or intolerant polycythemia vera in South Korea: one-year results from a phase 2 study

**DOI:** 10.1007/s44313-024-00026-6

**Published:** 2024-07-09

**Authors:** Seug Yun Yoon, Sung-Eun Lee

**Affiliations:** 1 Division of Hematology & Medical Oncology, Department of Internal Medicine, Soonchunhyang University Seoul Hospital, Seoul, Korea; 2 Department of Hematology, Seoul St. Mary’s Hospital, College of Medicine, The Catholic University of Korea, 222 Banpodae-ro, Seocho-Gu, Seoul, 06591 Korea

To the editor

Polycythemia vera (PV) is a Philadelphia chromosome-negative chronic myeloproliferative neoplasm (MPN) marked by elevated erythrocyte levels, an increased risk of thrombosis, and potential progression to myelofibrosis or acute myeloid leukemia (AML) [1, 2]. Hydroxyurea (HU) is extensively used for short-term disease management to reduce cell proliferation in patients needing cytoreductive therapy, following consensus guidelines from the European LeukemiaNet (ELN) and the National Comprehensive Cancer Network (NCCN) [3]. However, HU treatment presents several challenges, including side effects such as cytopenia, aphthous ulcers, leg ulcers, and the risk of secondary malignancies [4, 5]. Notably, about 20% of patients exhibit resistance or intolerance to HU [6], and when evaluated using the modified ELN criteria, this figure rises to approximately 40% [7]. For these patients, ruxolitinib or pegylated interferon is recommended. Yet, the implementation of these treatments is hindered in Korea due to a lack of reimbursement. Furthermore, most research on interferon in PV has focused on its use as an early-stage treatment option due to its disease-modifying properties [8, 9]. Consequently, data on the clinical efficacy and safety of interferon in patients resistant or intolerant to HU are sparse, particularly regarding treatment with ropeginterferon alfa-2b. At the 2023 European Hematology Association (EHA) meeting, interim results from a phase 2 Korean single-arm, open-label multicenter study were presented. This study compared the response to ropeginterferon alfa-2b in HU- naïve patients versus those resistant or intolerant to HU. The findings demonstrated that ropeginterferon alfa-2b effectively treats PV patients, regardless of prior HU therapy [10]. This study was the first to illustrate the effectiveness of ropeginterferon alfa-2b in PV patients with HU resistance or intolerance. As the study has now completed one year of treatment, we aim to provide an update on the one-year treatment outcomes in this patient group.

This original study aimed to evaluate the clinical and molecular responses to ropeginterferon alfa-2b and to explore the relationship between efficacy and molecular response, specifically through reductions in *JAK2*V617F allele burden. Enrollment was limited to patients requiring cytoreductive therapy, resulting in the inclusion of 46 HU-resistant or intolerant patients out of a total of 99. The determination of HU resistance or intolerance was guided by the modified ELN criteria. Ropeginterferon alfa-2b was administered subcutaneously every two weeks, starting at 500 mcg, with a dose escalation scheme that included 250 mcg, 350 mcg, and 500 mcg. If a 500 mcg dose proved intolerable, a reduction to dose level -1 (350 mcg) or -2 (250 mcg) was permitted based on the investigator’s judgment. This study received approval from the Korean Ministry of Food and Drug Safety and each local ethics committee.

For the purposes of this paper, 43 patients from the HU-resistant or intolerant subgroup were analyzed, as defined by the full analysis set. The median age of these patients was 59 years (range 27–81), with males comprising 53.5% of the cohort. Among the participants, 42% had hypertension and 16% had diabetes. Baseline characteristics are detailed in Table 1. Complete hematologic response (CHR) was defined according to ELN criteria category B, and Molecular Response (MR) was defined as a partial response based on the 2009 ELN response criteria.

**Table 1:** Demographics and baseline characteristics

**Hydroxyurea Resistance or Intolerance group**	***N* = 43**
**Age, years, median (range)**	59.0 (37–81)
**Sex, No. (%)**	
**Female**	20 (46.5)
**Male**	23 (53.5)
**PV diagnosis, months, median (range)**	73.96 (4.29–225.79)
**Risk stratification, No. (%)**	
**Low**	23 (53.5)
**High**	20 (46.5)
**Hypertension, No. (%)**	18(42)
**Diabetes, No. (%)**	7 (16)
**Hct (%), median (range)**	48.70 (45.10–60.40)
**Hgb (g/dl), median (range)**	15.8 (12.0–19.15)
**Platelets (10**^**9**^**/L), median (range)**	485 (162–1062)
**WBC (10**^**9**^**/L), median (range)**	12.89 (5.96–34.50)
**ANC (10**^**9**^**/L), median (range)**	10.58 (4.19–32.02)
**RBC (10**^**6**^**/L), median (range)**	5.73 (8.87–4.23)
***JAK2* V617F mutation (%), median (range)**	74.68 (0.44–97.17)

*Abbreviations*: *Hct* Hematocrit, *Hgb* Hemoglobin, *RBC* Red Blood Cell, *WBC* White Blood Cell, *ANC* Absolute Neutrophil Count

At 48 weeks, 19 out of 36 patients (52.8%) achieved a complete hematologic response (CHR), and 13 (39.4%) achieved a molecular response (MR), as shown in Table 2. The mean reduction in *JAK2*V617F allele burden was 16.95%, with a standard deviation of 21.34% at 48 weeks (Fig. 1). The proportion of patients achieving CHR and MR exhibited a gradually increasing trend throughout the treatment period. Regarding safety, 35 patients (76%) experienced at least one adverse event (AE) during the study. AEs considered related to the study treatment occurred in 24 patients (52%). The most common treatment-related AEs were hepatotoxicity, marked by elevated levels of aspartate aminotransferase (AST), alanine aminotransferase (ALT), and gamma-glutamyl transferase (GGT), affecting 12 patients (26%). Serious adverse events (SAEs) were reported in six patients (13%), including two non-treatment-related thrombotic events (one cerebral infarction and one hematoma). Additionally, two SAEs were assessed as related to the study drug (one case of chest pain and one of hepatotoxicity). Despite these incidents, no patient discontinued the study due to adverse events. This suggests that ropeginterferon alfa-2b is both effective and tolerable in patients with resistance or intolerance to HU.

**Table 2:** CHR and MR during the study period

**Visit**	**CHR (n/N, % (95% CI))**	**MR (n/N, % (95% CI))**
**12 weeks**	4/43, 9.3 (0.62–17.98)	7/42, 16.67 (5.40 – 27.94)
**24 weeks**	12/40, 30.0 (15.80 – 44.20)	8/38^a^, 21.05 (8.09 – 34.01)
**36 weeks**	17/38, 44.74 (28.93 – 60.55)	12/35^a^, 34.29 (18.56 – 50.02)
**48 weeks**	19/36, 52.78 (36.47 – 69.09)	13/33^a^, 39.39 (22.72 – 56.06)

*Abbreviations*: *CHR* Complete Hematologic Response, *MR* Molecular Response

^a^The sample has been excluded from the analysis due to a DNA concentration lower than 5 ng/uL

**Fig. 1:**
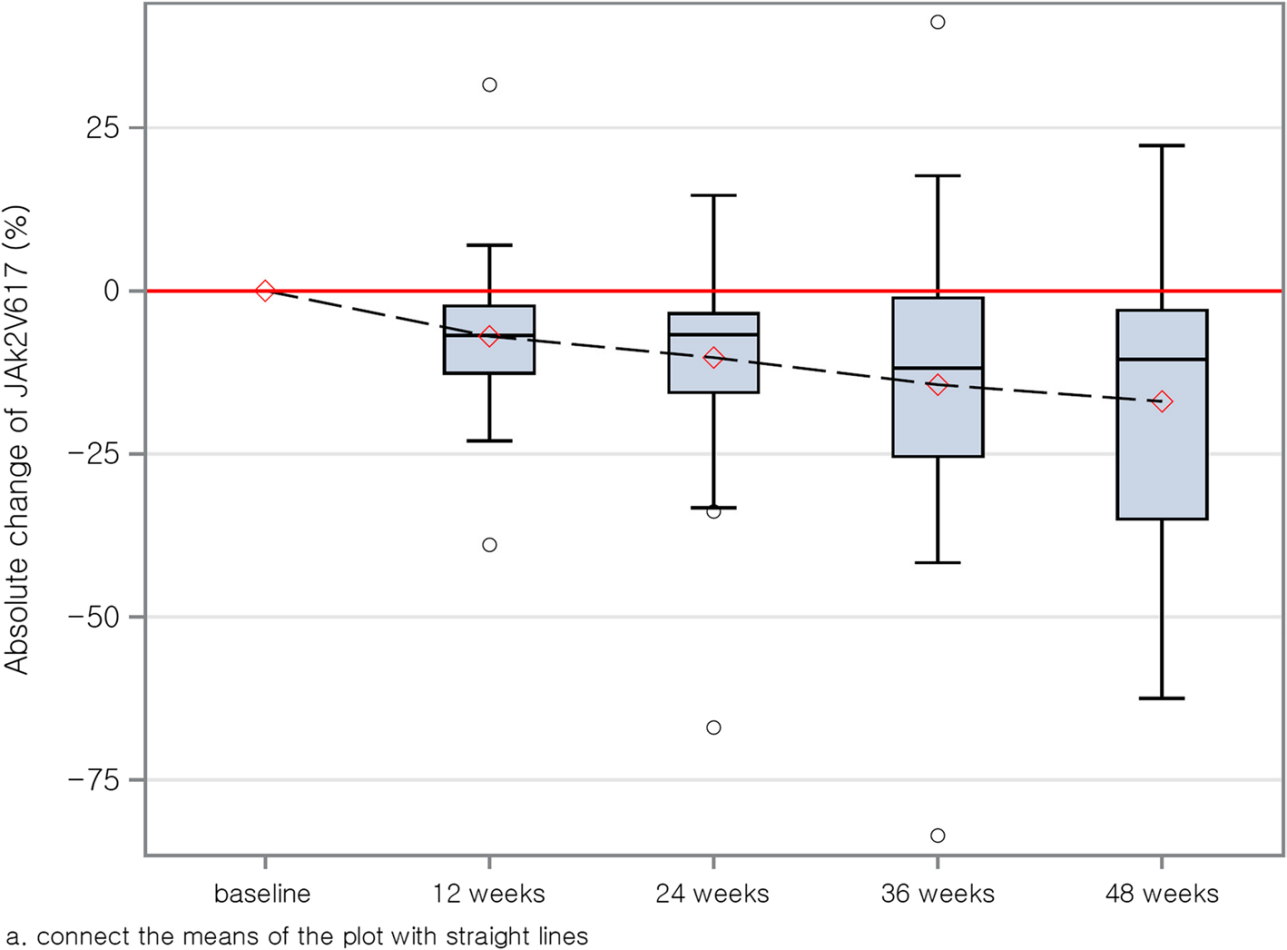
Absolute percentage change (%) in *JAK2*V617F allele burden from baseline

However, the study has limitations. It lacks detailed data on previous HU therapy, such as the maximum tolerated dose and specific reasons for intolerance. Furthermore, according to recent studies [6], patients with HU resistance demonstrate a higher incidence of disease progression and poorer survival outcomes compared to those with HU intolerance. This indicates a need for a more nuanced analysis based on patients’ prior experiences with HU. Additionally, the absence of long-term follow-up data beyond 48 weeks limits our understanding of the prolonged effects of ropeginterferon alfa-2b, its impact on disease progression, and patient outcomes. Ongoing studies are expected to provide insights into the sustainability of treatment responses.

This result offers compelling evidence of ropeginterferon alfa-2b's efficacy in stabilizing hematologic parameters and reducing *JAK2*V617F allele burden in PV patients with resistance or intolerance to HU. It provides valuable insights into a treatment strategy for patients with limited options. While anticipating novel treatment options for HU-resistant or intolerant PV, further research is essential to address this study's limitations and to validate the long-term efficacy and safety of ropeginterferon alfa-2b.

## Data Availability

No datasets were generated or analysed during the current study.
